# Remediation of wastewater by biosynthesized manganese oxide nanoparticles and its effects on development of wheat seedlings

**DOI:** 10.3389/fpls.2023.1263813

**Published:** 2023-12-06

**Authors:** Aneeza Ishfaq, Muhammad Shahid, Muhammad Nawaz, Danish Ibrar, Sabir Hussain, Tanvir Shahzad, Faisal Mahmood, Afroz Rais, Safia Gul, Abdel-Rhman Z. Gaafar, Mohamed S. Hodhod, Shahbaz Khan

**Affiliations:** ^1^ Department of Environmental Sciences & Engineering, Government College University, Faisalabad, Pakistan; ^2^ Department of Bioinformatics & Biotechnology, Government College University, Faisalabad, Pakistan; ^3^ Department of Agricultural Engineering, Khwaja Fareed University of Engineering and Information Technology, Rahim Yar Khan, Pakistan; ^4^ Crop Science Institute, National Agricultural Research Centre, Islamabad, Pakistan; ^5^ Department of Botany, Sardar Bahadur Khan Women’s University, Quetta, Pakistan; ^6^ Department of Botany and Microbiology, College of Science, King Saud University, Riyadh, Saudi Arabia; ^7^ Faculty of Biotechnology, October University for Modern Sciences & Arts, 6th October, Egypt; ^8^ Colorado Water Center, Colorado State University, Fort Collins, CO, United States

**Keywords:** microbial synthesis, photocatalytic degradation, antioxidants activities, wastewater treatment, crop cultivation

## Abstract

**Introduction:**

Nanoparticles play a vital role in environmental remediation on a global scale. In recent years, there has been an increasing demand to utilize nanoparticles in wastewater treatment due to their remarkable physiochemical properties.

**Methods:**

In the current study, manganese oxide nanoparticles (MnO-NPs) were synthesized from the *Bacillus flexus* strain and characterized by UV/Vis spectroscopy, X-ray diffraction, scanning electron microscopy, and Fourier transform infrared spectroscopy.

**Results:**

The objective of this study was to evaluate the potential of biosynthesized MnO-NPs to treat wastewater. Results showed the photocatalytic degradation and adsorption potential of MnO-NPs for chemical oxygen demand, sulfate, and phosphate were 79%, 64%, and 64.5%, respectively, depicting the potential of MnO-NPs to effectively reduce pollutants in wastewater. The treated wastewater was further utilized for the cultivation of wheat seedlings through a pot experiment. It was observed that the application of treated wastewater showed a significant increase in growth, physiological, and antioxidant attributes. However, the application of treated wastewater led to a significant decrease in oxidative stress by 40%.

**Discussion:**

It can be concluded that the application of MnO-NPs is a promising choice to treat wastewater as it has the potential to enhance the growth, physiological, and antioxidant activities of wheat seedlings.

## Introduction

Nanotechnology, the broad discipline of the twenty-first century, has a remarkable influence on global economic success and the commercial sector. Utilization of nanotechnology is increasing day by day in the agriculture field, which involves cultivation, harvesting, processing, storage, packaging, and transportation of agricultural products. Nano-techniques have shown significant potential to improve food quality and agriculture products (Noman et al., 2020; Kaushik, 2021). A nanoparticle is a small particle that ranges from 1 to 100 nanometers in size with distinctive physical and chemical properties (Korbekandi et al., 2012). The nanoparticles, owing to the unique features, have gained the attention of scientists for use in different fields of life (Islam et al., 2018; Sharma et al., 2019), including medicine, wastewater treatment, agriculture, energy products, and the remediation of different environmental pollutants (Duhan et al., 2017; Singh et al., 2018; Noman et al., 2020; Zhang et al., 2020). For the treatment of wastewater, a range of techniques have been employed, including nanotechnology. Nano-photocatalytic technology has received a lot of attention in the field of water pollution prevention.

Various techniques, including physical, chemical, and biological, are considered for the synthesis of nanoparticles. Physical and chemical methods demand difficult conditions like high temperature and pressure, very costly equipment, large space to set up large machinery, and the use of toxic chemicals (Hoseinpour and Ghaemi, 2018). Numerous chemical and physical techniques use toxic substances that can have adverse effects on the environment. Consequently, there is a dire need to explore environmentally friendly and sustainable approaches. Researchers are investigating the potential of various biological entities, such as bacteria, fungi, higher plants, actinomycetes, and viruses, for the synthesis of nanoparticles (Agrawal et al., 2022). However, biological methods to synthesize nanoparticles are getting popularity as they are cost-effective and environmentally friendly (Castro et al., 2014). Numerous bacterial and fungal strains are recognized for their ability to synthesize nanoparticles. Bacteria, with their distinctive quality of metal reduction, play a vital role in the synthesis of nanoparticles. Their resistance to acute environmental conditions, including high temperature and pressure, makes them well-suited for the synthesis of nanoparticles (Das et al., 2014). Different microbial strains, such as *Pseudomonas stutzeri, Morganella* sp, *Plectonema boryanum*, *Thermomonospora* sp, *Rhodococcus* sp, and *Pseudomonas aeruginosa*, have been effectively employed in the biosynthesis of nanoparticles (Thakkar et al., 2010; Idris and Yuliar, 2022).

Micronutrients are required in very small quantities by the plant and can affect indirectly or directly photosynthesis and play a vital role in plant protein synthesis, respiration, and the reproductive system. Manganese (Mn) is a micronutrient that plays a crucial role in various plant functions, including respiration and photosynthesis. Nanoscale Mn is reported to be less effective in inhibiting plant growth physiological processes and more efficient at reducing abiotic stressors in plants than traditional bulk or ionic Mn molecules (Ye et al., 2019). Manganese nanoparticles are widely used in industry for a variety of purposes, including supercapacitors, catalysis, biosensors, ion sieves, molecular adsorption, high-density magnetic storage media, batteries, drug delivery systems, and magnetic resonance imaging (Aronson et al., 2000; Ammundsen and Paulsen, 2001). In recent studies, manganese oxide nanomaterials even outperformed MCM-22 and red mud in terms of their ability to adsorb a methylene blue dye (Chen and He, 2008). Furthermore, it was further shown that a hierarchical MnO_2_-coated magnetic nanostructure displayed a significantly greater capacity for adsorption than pure magnetic nanoparticles (Kim et al., 2013).

Due to the limited availability of water sources in developing countries, wastewater is being used for agriculture near fields around industrial premises for growing vegetable crops (Roy and Edwards, 2019). Emerging pollutants like pesticides, chemical fertilizers, and other chemical substances such as dyes (Benkhaya et al., 2020; Slama et al., 2021), heavy metals (Engwa et al., 2019; Shrestha et al., 2021), hormones, beauty products, detergents, and drugs enter water supply systems and can either directly or indirectly damage human health. However, the presence of potentially hazardous substances (PTEs) is the primary issue with wastewater used for the irrigation of crops. The accumulation of PTEs in the soil and crops caused by wastewater irrigation had negative impacts not only on soil fertility but also on human beings (Qadir et al., 2010; Azimi et al., 2017). When plants are irrigated with wastewater, the elevated concentrations of sulfate and phosphate lead to accumulation within the plants. Plants require an optimal balance of nutrients, including nitrate, sulfate, and phosphate. The heightened levels cause a nutritional imbalance and disrupt the plant metabolism (Banon et al., 2011; Wardah et al., 2022). Agriculture serves as the foundation of the economy and is widely recognized as the primary source of income for rural people. According to Agricultural Organizations and Food organizations, the overall population of the world would increase by 9 to 10 billion by the year 2050 (Jaramillo and Restrepo, 2017). There is a need to increase food productivity by 25% to 70% to feed the growing population. Wheat (*Triticum aestivum L*.) is a staple food in many countries including, Pakistan, and an important part of the diet. One-third of the world’s population gets their nutritional needs met through wheat, which also has more nutritional value than other cereals.

According to our observation and knowledge, many studies were mentioned before synthesizing MnO-NPs from plants and chemically checking their potential for wastewater treatment, but not a single study on the production of manganese nanoparticles from *Bacillus flexus* and then application to wastewater treatment. Similarly, using *B. flexus* to produce MnO-NPs and then applying them to wastewater might be a new biosource. Based on the above-mentioned findings, we hypothesized that biosynthesized MnO-NPs may have a beneficial potential to remove contaminants from wastewater. In the current study, the synthesis of MnO-NPs was accomplished using pre-isolated *B. flexus* to treat the wastewater. Furthermore, the treated wastewater was applied to wheat seedlings to explore the potential of treated wastewater for agricultural purposes. In addition, the performance of wheat seedlings regarding physical growth, and physiochemical parameters were also studied under the impact of untreated and treated wastewater, and distilled water as irrigational water.

## Materials and methods

### Biosynthesis of manganese oxide nanoparticles by *B. flexus*

**Figure 1:**
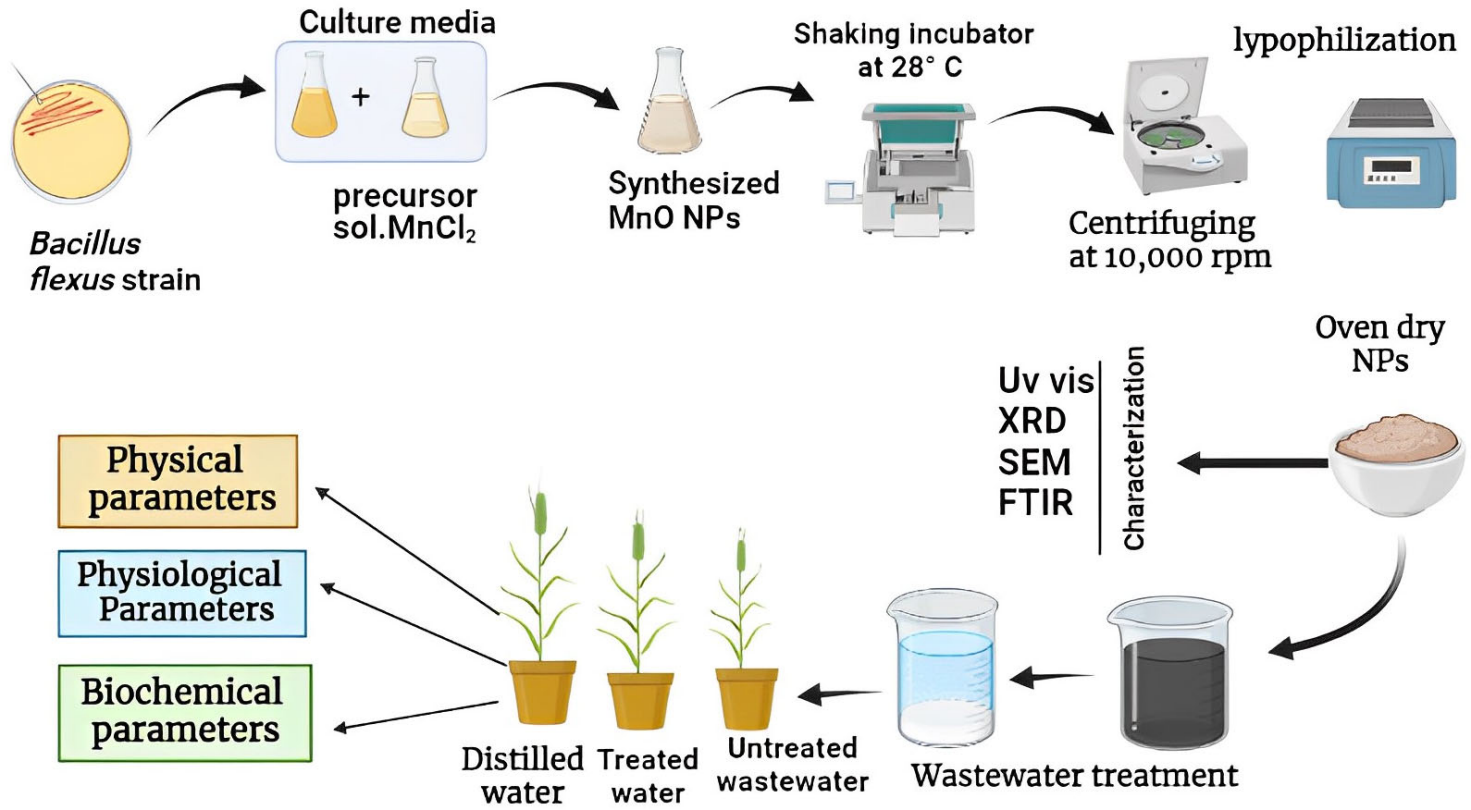
A schematic diagram for biologically synthesizing of manganese oxide nanoparticles (MnO-NPs) and their application on wheat seedlings.

Pre-isolated and identified bacterial strain of *B. flexus* was obtained from Environmental Microbiology Laboratory, Department of Environmental Sciences, Government College University Faisalabad, Pakistan. Manganese oxide nanoparticles (MnO-NPs) were synthesized by using the strain of *B. flexus* at 10mM concentration of manganese dichloride tetrahydrate (MnCl2.4H2O) salt (Sigma-Aldrich CAS number: 13446-34-9) according to the methodology developed by Wright et al. (2016). The bacterial strain was inoculated in 100mL of nutrient broth and then placed in an orbital rotary shaker at 120rpm (28°C) for 24 hours. After 24 hours of rotation, 0.1 Mm of manganese chloride was added to the culture which changed color from yellow to dark brown. The culture was again placed for the further 24 hours in a rotary shaker and the pallet was collected by centrifuging at 700 rpm for 10 minutes. The resulting pellet was kept in the oven for 24 hours to get a fine dry powder of MnO-NPs. A schematic protocol for biologically synthesizing MnO-NPs and their application on wheat seedlings is presented in **Figure 1**.

### Characterizations of manganese oxide nanoparticles

The morphology of MnO-NPs was determined by the scanning electron microscope (SEM LEO 1530, Germany) by following the procedure of Vladár and Hodoroaba (2020). The crystalline structure of manganese oxide nanoparticles was examined by X-ray diffraction (XRD) analysis (PANalytic X PERTPRO, USA) in 20-80°C (2θ), 45KV. The formation of manganese nanoparticles was determined by using a double-beam UV-VIS spectrophotometer (UH 5300, Hitachi Japan) at 300-500 nm ranges. The functional groups and associated proteins were determined by using a Fourier transmission electron microscope (FTIR-Bruker TENSOR-27) by adopting the procedure of Baraton and Merhari (2007). FTIR analysis was performed on the fine powder for MnO-NPs of bacterial strains at a scanning frequency of 32 with a range of 350-4000 cm^-1^.

### Application of manganese oxide nanoparticles to treat wastewater

#### Sample collection and treatment of the wastewater

The wastewater samples were collected in sterilized bottles from the drain originated from industrial state, Mohalla Tahir, Jhang Road, Faisalabad, Pakistan. Collected samples were transferred to the Environmental Microbiology Laboratory, Department of Environmental Sciences, Government College University Faisalabad, Pakistan, for further analysis. Wastewater samples were centrifuged to remove particulate matter. To check the photocatalytic degradation and adsorption potential, 1 g of MnO-NPs were added in 1000 ml of wastewater. Three replicates of the sample were vortexed and incubated under sunlight with their respective controls. After 7 hours of incubation, the samples were centrifuged at 9000 rpm for 10 minutes to remove the suspended nanoparticles. The pH, EC, COD, sulfates, phosphates, and color intensity were determined before and after wastewater treatment by following the procedure of De Feo and Ferrara (2017).

### Application of untreated and treated wastewater on wheat seedlings

#### Experimental location and crop husbandry

A pot experiment was conducted to explore the impact of treated and untreated wastewater on the wheat (cultivar Akbar-2019) crop during season 2022-2023 in the Department of Environment Sciences, Government College University Faisalabad, Pakistan (31.4°N, 73.06°E). Fresh seeds of wheat (*Triticum aestivum* L.) were collected from the Ayub Agriculture Research Institute, Faisalabad, Pakistan. The seeds of wheat were washed with distilled water and sterilized five times with 5% of sodium hypochlorite solution. Seeds were then air-dried at room temperature. Rhizosphere soil, 0-15 cm, was taken in triplicate with the augur from Ayub Agricultural Research Institute, Faisalabad, Pakistan. Soil samples were taken randomly throughout the agriculture field and stored in polythene bags. Each soil sample was mixed thoroughly to get uniform composite samples of homogenous nature having organic matter, pH, and EC of 0.72%, 2.7, and 7.8 dS m^-1^, respectively. The concentrations of nitrogen, phosphorus and potassium in the soil were 0.64%, 11.7 ppm and 158 ppm, respectively.

The experiment was laid down in a completely randomized design (CRD) with three replications. Each pot was filled with 1 kg of soil and 8 seeds of wheat were sown in each pot. The treatments, wastewater, treated wastewater, and distilled water, were applied at sowing time and once a week for a period of 30 days. In total, 50 ml of water, encompassing all treatments, were utilized over the course of experimentation. After the germination of seeds, Hoagland nutrient solution was given to each pot and the germination rate was observed at days three, six, and nine. By the completion of germination, after every two days modified half-strength Hoagland’s nutrient solution (Hoagland and Arnon, 1950) was used to irrigate the pots throughout the experiment. Hoagland solution was provided as an alternative to fertilizer to ensure consistent nutrient availability, enhancing experimental reproducibility, and minimizing variability due to nutrient deficiencies.

After 30 days of plantation, the plants were harvested. Plant samples were cleaned with deionized water to remove aerial deposits. The shoots and roots of the plants were separated. The fresh weights of the harvested shoots and roots were recorded. These samples were then stored in falcon tubes at 4°C in a refrigerator for further analysis.

### Determination of growth parameters

The physical growth parameters of shoot length, root length, shoot fresh weight, root fresh weight, shoot dry weight, root dry weight, and germination rate of plants were determined by using the methodology given by Noman et al. (2020). The ratio of germinated seeds to the overall number of seeds in the pot was used to measure the germination rate. When a radical sprout 1 mm in length emerged from the seed, the seeds were considered germinated. The following equation was used to calculate the germination rate.


Seed Gemination(%)=Number of germinated seeds/Total number of seeds×100

### Photosynthetic pigments analysis

Using the Arnon (1949) method, 0.1g fresh wheat leaves were ground in a mortar pestle using an ice tub to avoid degradation of plant leaves into 80% acetone (80ml acetone + 20ml D.w) and were kept overnight at -4°C in the refrigerator. The enzyme extract was then centrifuged at 10000 RPM for 10 minutes. The spectrophotometer was used to measure the absorbance of the supernatant at three different wavelengths: 645, 480, and 663 nm. The chlorophyll content was determined as mg g-1 FW. The amount of chlorophyll and carotenoid was determined by using the following formula.


Total Chlorophyll=[20.2(OD 645)−8.02(OD 663)×V/10000×W]

Chlorophyll a=[12.7(OD 663−2.69(OD 645)×V/10000×W]

Chlorophyll b=[22.9(OD 645−4.68(OD 663)×V/10000×W]

Carotenoids=[(OD 480)+0.114(OD 663)−0.638(OD 645)]/2500

Where,

OD = Optical density, V = volume of extract (ml), and W = weight of fresh leave (g)

### Determination of mineral elements

To determine the concentrations of nutrient elements like Zn, Fe, Mn, and Cu in plants roots and shoots, the plant root and shoot dry biomass of 0.5 g was placed into a flask, and then concentrated acids (HClO4-HNO3) were added in a 1:3 ratio. The mixture was allowed to keep for 24 hours. Subsequently, the solution was subjected to digestion on a hot plate, heating it until it became colorless. Deionized water was added to bring the total volume to 50 ml. A control solution without a sample was maintained to ensure the accuracy of the sample results. The Zn, Fe, Mn, and Cu concentrations were determined using atomic absorption spectroscopy (Hitachi, Model 7JO-8024; Tokyo, Japan) (Bhat et al., 2010).

### Determination of oxidative stress markers and antioxidants

#### Malondialdehyde

For the estimation of malondialdehyde (MDA), the method of Young and Trimble (1991) was followed. To measure the amount of lipid peroxidation of fresh leaf samples, the thiobarbituric acid (TBA) assay was used. 0.1 g of the plant’s fresh leaf was powdered in 5 mL of 5% (w/v) TCA (trichloroacetic acid). The extract was then centrifuged for 10 minutes at 12000 x g. Thiobarbituric acid (TBA) 0.5% (W/v) in 20% TCA was pipetted into 4 mL of supernatant (trichloroacetic acid). The resulting mixture is then cooled inside an ice bath at 95°C for 30 minutes. The solution was then vortexed for 5 minutes and then measured the absorbance of the mixture at two wavelengths of 532 and 600 nm.

#### Hydrogen peroxide

Hyslop et al. (1995) technique was used to calculate the hydrogen peroxide (H_2_O_2)_ concentration of a sample. The 5 ml of 0.1 percent (w/v) trichloroacetic acid was added to a 0.1 g fresh plant sample in the ice tub for grinding. 1 ml of KI was added into 0.5 ml of potassium phosphate buffer and then 0.5 ml of supernatant was added, and the sample was centrifuged. After the centrifugation, the sample was left for 10 minutes at room temperature. The absorbance was recorded at 390 nm by using a spectrophotometer. The H_2_O_2_ content was determined by using a comparison with the standard curve and distinctive H_2_O_2_ concentrations.

#### Ascorbate peroxidase

For the estimation of ascorbate peroxidase activity in wheat plants, the method given by Amako et al. (1994) was followed. 1 ml reaction mixture consisting of 50 mM phosphate buffer (pH 7.6), 0.1 mM Na-EDTA, 12 mM H_2_O_2_, and 0.25 mM ascorbic acid was mixed thoroughly. The test tubes were covered with aluminum foil to prevent light. A spectrophotometer was used to measure the absorbance of this solution at a wavelength of 290nm after every 20 seconds.

#### Catalase and peroxidase

The method described by Chance and Maehly (1955) and Cohen et al. (1996) was used to determine catalase (CAT) and peroxidase (POD) activities. By following this method, a 3 ml reaction mixture including 50 mM phosphate buffer (pH 7), and 0.1 mL pure enzyme extract, with 5.9 mM H_2_O_2_ was prepared. The standard CAT activity was calculated as an absorption shift of 0.01 unit over one minute. Enzyme extract was added to start the reaction and the mixture absorbance was calculated every 20 seconds and changes in the measurement at a frequency of 240 nm were noted. POD was measured using a reaction mixture of 3 mL (20 mM guaiacol, 50 mM phosphate buffer of pH 5, 50 mM H_2_O_2_, and 0.1 mL enzyme extract fresh leaves extract). The absorbance was measured at 470 nanometers of wavelength.

#### Superoxide dismutase

For the estimation of superoxide dismutase (SOD), the methodology of Beauchamp and Fridovich (1971) was adopted. For this purpose, a 3ml reaction solution was prepared by adding (50 µl riboflavin, 50 µl nitro blue tetrazolium (NBT), 100 µl L-methionine, 50 µl enzyme extract, 100 µl triton-X, 400 µl H_2_O, and 250 µl). After mixing, the reaction mixture was added into a test tube. These test tubes were exposed to light for 10-15 minutes. At the wavelength of 560 nm, the absorbance of this solution was recorded using a spectrophotometer.

### Statistical analysis

A statistical package “Statistix 8.1” was used to analyze the collected data of various parameters. The trail was conducted under a complete randomized design (CRD) arrangement. One-way ANOVA (analysis of variance) was to observe the significance of treatment through t-test. Fisher’s least significant difference test, with a *p* ≤ 0.05, was used to compare the treatments’ means.

## Results

### Characterization of manganese oxide nanoparticles

#### Ultraviolet-visible spectrophotometer analysis

**Figure 2:**
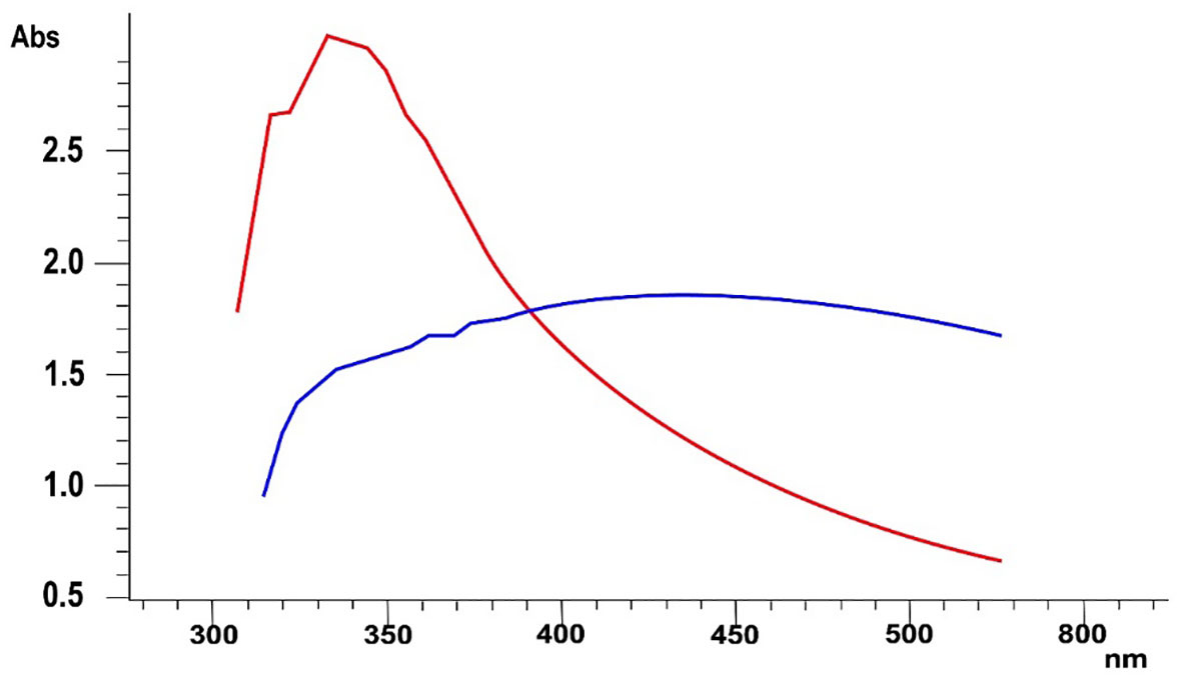
The UV-visible spectroscopy of biosynthesized manganese nanoparticles using *Bacillus flexus*. The blue line indicates the source salt utilized in the nanoparticle synthesis process. Red line highlights the presence of nanoparticles.

The culture of *B. flexus* was incubated at optimum conditions, i.e., at 28°C for 24 hours after the addition of 10 mM MnCl salt solution. After 24-hour incubation, it was observed that the color of the reaction mixture was changed from light yellow to dark brown. A small quantity of the reaction mixture was subjected to an ultraviolet-visible spectrophotometer. **Figure 2** depicts a peak at 325.23 nm which is an indication of the biosynthesis of MnO-NPs. The UV spectroscopy ranges for manganese oxide nanoparticles typically span from approximately 200 to 400 nanometers (nm). Redshift peak shifts shorter that showed small size of nanoparticles.

### X-ray diffraction analysis

**Figure 3:**
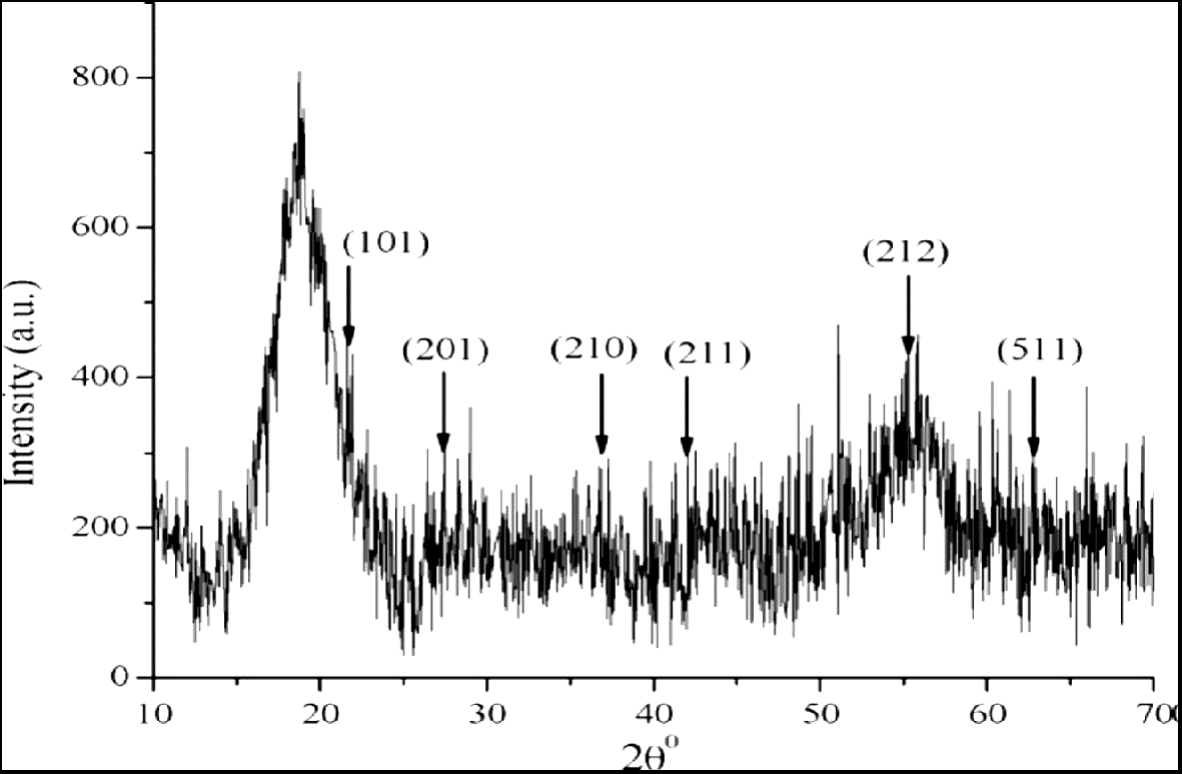
X-ray diffraction (XRD) pattern of biosynthesized manganese nanoparticles (MnO-NPs) produced by *Bacillus flexus*: Characterizing crystal structure and composition.


The X-ray diffraction pattern indicates the crystalline nature of the nanoparticles. The analysis using MnO-NPs from strain *B. flexus* showed a peak position of 2θ ranging from 10θ to 70θ at 22.79°, 27.79°, 38.20°, 42.90°, 55.10°, and 64.50°, which were assigned to the (101), (201), (210), (211), (212) and (511). These peaks were according to the data of the standard diffraction value of MnO-NPs (JCPDS # 39-0375) indicating the crystalline nature of nanoparticles using the Debye-Sherer’s equation (**Figure 3**).

D=Kλ/βcosθ

Where,

D = size of the crystallite, k = shape factor, λ = wavelength of radiation.

The average crystallite size in the samples using Scherrer’s equation was less 41 nm for MnO nanoparticles.

### Scanning electron microscopy

**Figure 4:**
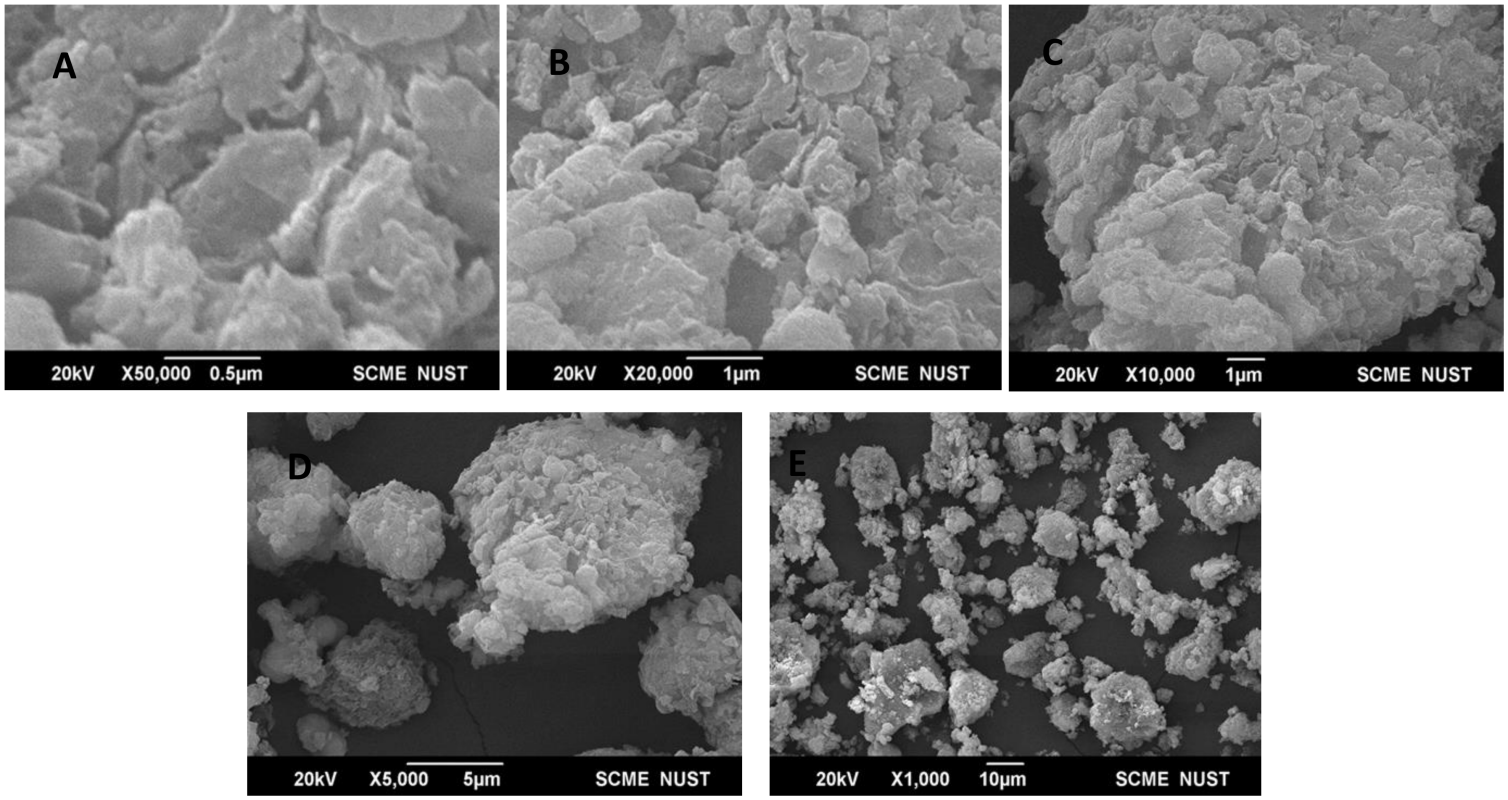
Scanning electron microscopy (SEM) images of biosynthesized manganese nanoparticles at various magnifications: **(A)** (at 0.5 micrometers), **(B)** (at 1 micrometer), **(C)** (at 1 micrometer), **(D)** (at 5 micrometers), **(E)** (at 10 micrometers).

Scanning electron microscopy (SEM) analysis provides the details about the surface morphology of MnO-NPs biosynthesized from bacterial strains of *B. flexus*. From the SEM micrograph, at different magnification scales ranging from 1000X-50,000X, the SEM images of MnO-NPs were collected and analyzed. The resulting data showed that the size of the nanoparticle ranged from 21-30 nm. Furthermore, agglomeration is also observed between the large-size particles, which indicates that different capping proteins and structures were required to stabilize the small-size particles. The SEM data of biogenic MnO-NPs from *B. flexus* is shown in **Figure 4**.

### Fourier-transform infrared spectroscopy

**Figure 5:**
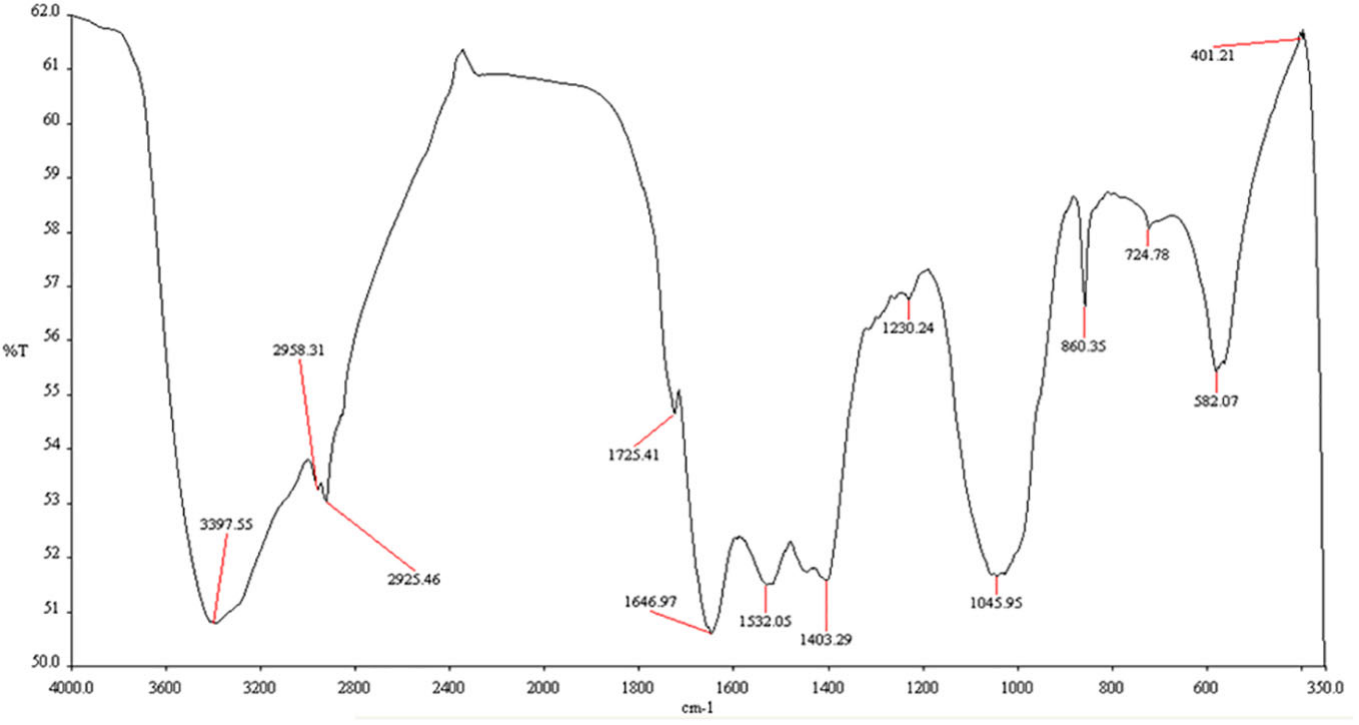
Fourier-transform infrared spectroscopy (FTIR) spectrum of biosynthesized manganese nanoparticles using *Bacillus flexus*.

The Fourier-transform infrared spectroscopy (FTIR) analysis investigates the surface characteristics of biosynthesized MnO-NPs. The surface characteristics of biogenic manganese nanoparticles were explored by FTIR analysis in the wave ranging from 350-4000 cm^-1^ (**Figure 5**). The presence of bands at 401.21 showed the probability of the presence of MnO. The FTIR spectra of MnO-NPs produced from *B. flexus* showed absorption peaks at 401.21, 582.07, 724.78, 860.35, 1045.95, 1230.24, 1403.29, 1532.05, 1646.97, 1725.41, 2925.46, 2958.31 and 3397.55 cm^-1^. The strongest peak at 3397.55 cm^-1^ was observed due to the broad bonded N-H/O-H stretching group of alcohol and amine. The peaks at 1725.41 cm^-1^ and 1532.05 cm^-1^ represented the C-O group of aldehydes and N-O stretching group of nitro compounds, respectively. The peaks at 1230.24 cm^-1^ were due to the C-O group of ether stretching.

### Wastewater treatment by manganese oxide nanoparticles

The analysis of untreated wastewater revealed the values of pH and EC, the concentrations of COD TDS, sulfates and phosphates that exceeded the permissible limits set by the Environmental Protection Agency (EPA). These parameters were found to be significantly higher than the regulatory thresholds established by the EPA. Overall, the concentrations of all these indicators were significantly decreased in the industrial effluents, which were treated with MnO-NPs. **Table 1** shows the parameter values before and after treatments with MnO-NPs.

**Table 1:** Pollutants reduction in industrial wastewater by MnO-NPs and comparison with National Environmental Quality Standards (NEQS).

Parameters	Wastewater	Treated wastewater	Removal %	NEQS limits
Color Removal	0.13 ± 0.002	0.03 ± 0.004	77.05	NG
EC (dSm^-1^)	5.33 ± 0.20	3 ± 0.60	—	NG
pH	8.7 ± 0.11	7.3 ± 0.05	—–	6.5-8.5
COD (mg L^-1^)	378 ± 3.8	78 ± 0.46	79	200
TDS (mg L^-1^)	3417 ± 5.7	2147 ± 45	37	1000
Sulfates (ppm)	767 ± 17	275 ± 32	64	500
Phosphates (ppm)	354 ± 3.84	125 ± 36	64.5	NG

Each value presented is the average of three replicates, accompanied by the standards deviation. It is important to note that NEQS of Pakistan government the permissible limits for industrial effluents discharge.

NEQS, National Environmental Quality Standards; EC, Electrical conductivity; COD, chemical oxygen demand; TDS, total dissolved solids; NG, not given.

### Seedlings growth attributes

**Figure 6:**
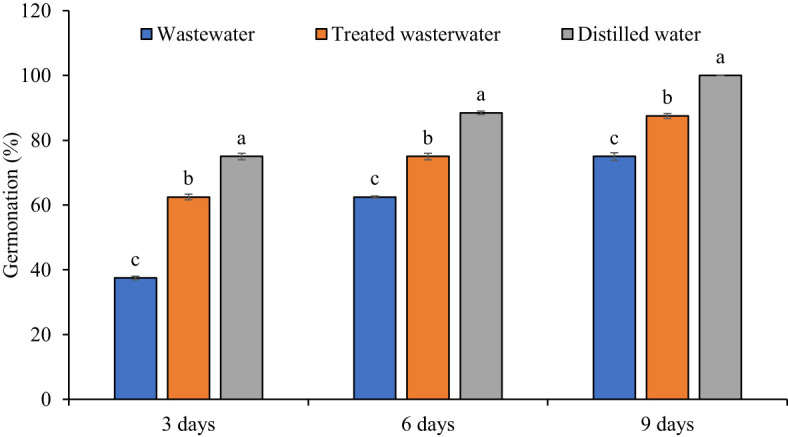
Impact of wastewater, treated wastewater, and distilled water on germination percentage at 3, 6 and 9 days after sowing of wheat seeds. The bars with different letters indicate significant differences from each other (*p*<0.05).

**Figure 7:**
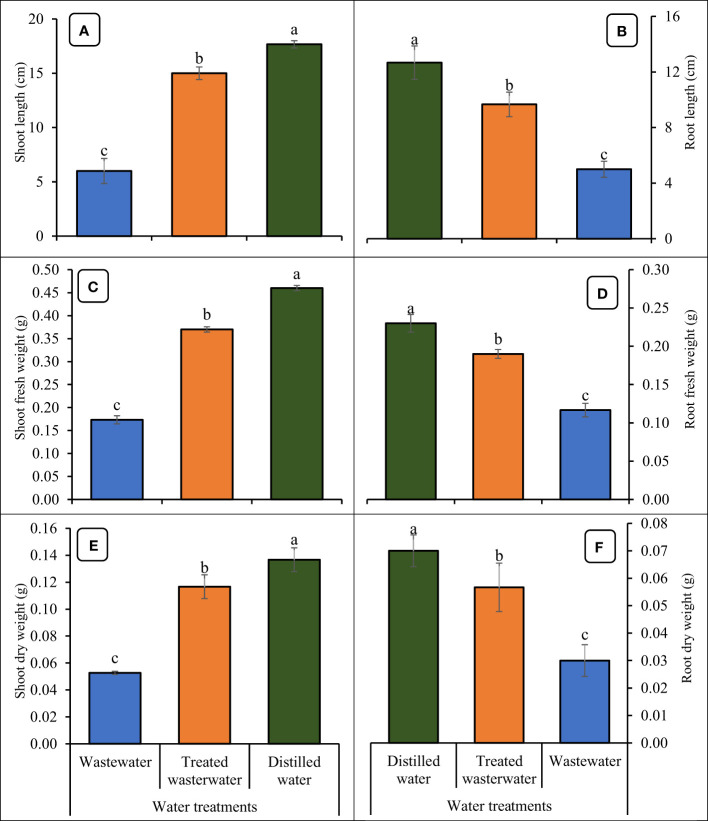
Impact of wastewater, treated wastewater, and distilled water on shoot length **(A)**, root length **(B)**, shoot fresh weight **(C)**, root fresh weight **(D)**, shoot dry weight **(E)**, and root dry weight **(F)** of wheat seedlings. The bars with different letters indicate significant differences from each other (*p*<0.05).

The growth parameters of the wheat seedlings increased significantly (*p ≤* 0.05) in treated wastewater as compared to untreated wastewater shown in **Figures 6**, **7**. The germination rate in wheat increased by 40% in treated wastewater treatment in comparison with untreated wastewater. On day six, germination rate increase was 16.6% in treated wastewater treatment in comparison with untreated wastewater (**Figure 6**). On day nine, the germination rate increased by 14.6% in treated wastewater in comparison with untreated wastewater treatment. Moreover, plants showed a significant increase in root and shoot length of 48% and 33% in treated wastewater, while a slight increase of 23% and 15% was recorded when compared to distilled water as compared to treated wastewater. According to statistical analysis, the fresh and dry shoot weights were increased by 42% and 26% respectively in treated wastewater as compared to untreated wastewater; however, these parameters recorded an 8% and 13% increase in distilled water as compared to treated wastewater. Similarly, fresh root and dry root weights were increased by 37% and 19% respectively in treated wastewater as compared to untreated; however, these parameters recorded a 13% and 9.6% increase in distilled water (control) as compared to treated wastewater (**Figure 7**).

### Minerals elements

**Table 2** showed the variations in mineral concentration in root and shoot of seedlings under wastewater, treated wastewater and distilled water. Zn and Mn uptake was notably higher in wheat seedling irrigated with treated wastewater compared to distilled water. Similarly, Cu and Fe uptake was notably higher in the wheat seedling irrigated with wastewater compared to distilled water.

**Table 2:** Impact of wastewater, treated wastewater, and distilled water on nutrient accumulation in root and shoot of wheat seedlings (n = 3).

Treatments	Zn (mg g^-1^)		Fe (mg g^-1^)		Mn (mg g^-1^)		Cu (mg g^-1^)	
	Root	Shoot	Root	Shoot	Root	Shoot	Root	Shoot
Wastewater	71.67 ^a^	69.00 ^a^	37.33 ^a^	36.00 ^a^	53.33 ^b^	52.33 ^b^	21.33 ^a^	18.67 ^a^
Treated wastewater	68.00 ^b^	67.00 ^a^	32.00 ^b^	33.00 ^b^	57.67 ^a^	54.33 ^a^	18.33 ^b^	16.33 ^b^
Distilled water	28.67 ^c^	4.33 ^b^	16.33 ^c^	15.33 ^c^	33.67 ^c^	29.00 ^c^	14.33 ^c^	12.67 ^c^
LSD	1.883	2.106	1.489	1.762	2.703	1.492	1.153	1.078

Fisher’s least significant difference test, with a p ≤ 0.05, was used to compare the treatments’ means. Values are means ± standard errors. Means sharing the same alphabets did not differ significantly.

### Physiological parameters

**Figure 8:**
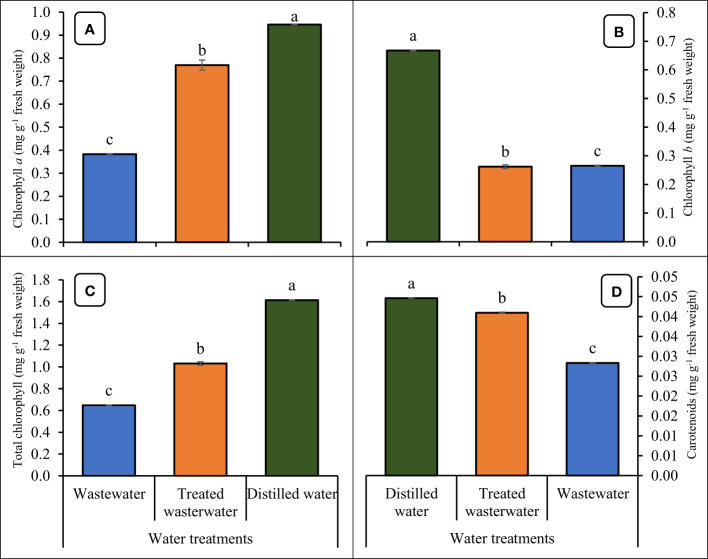
Impact of wastewater, treated wastewater, and distilled water on chlorophyll *a* content **(A)**, chlorophyll *b* content, total chlorophyll contents **(C)** and carotenoids **(D)** of wheat seedlings. The bars with different letters indicate significant differences from each other (*p*<0.05).

The chlorophyll content (Chlorophyll *a* and *b*), total chlorophyll, and carotenoids of the wheat crop led to a significant (*p ≤* 0.05) increase by the application of treated wastewater (**Figure 8**). Chlorophyll *a*, *b*, and total chlorophyll and carotenoids content of wheat were increased by 49.5%, 34.6%, 40.8%, and 40.2% in treated wastewater as compared to untreated wastewater respectively. Moreover, when we compared all these parameters recorded a slight increase of 18%, 13%, 21.8%, and 22.7% respectively in distilled water as compared to treated wastewater.

### Antioxidants and oxidative stress markers

**Figure 9:**
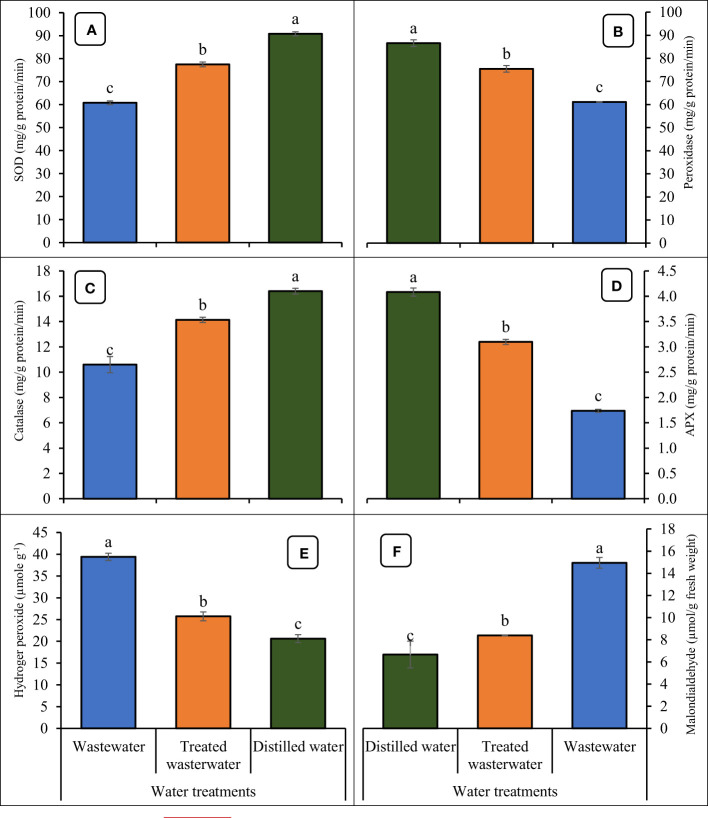
Impact of wastewater, treated wastewater, and distilled water on super oxide dismutase **(A)**, peroxide dismutase **(B)**, catalase **(C)**, ascorbate peroxidase **(D)**, hydrogen peroxide **(E)** and malondialdehyde **(F)** of wheat seedlings. The bars with different letters indicate significant differences from each other (*p*<0.05).

The application of treated wastewater led to an increase in the levels of antioxidants like super oxide dismutase (SOD), peroxidase (POD), catalase (CAT), and ascorbate peroxidase (APX) in wheat seedlings, as depicted in **Figure 9**. In treated wastewater, antioxidants like SOD, POD, CAT, and APX of wheat were increased significantly by 21%, 23%, 25%, and 43% as compared to untreated wastewater. Data regarding oxidative stress parameters are presented in **Figure 9**. The application of treated wastewater significantly decreased the level of H_2_O_2_ and MDA in wheat. In treated wastewater, H_2_O_2_ and MDA activities decreased by 43%, and 50% respectively, compared to untreated wastewater. However, in distilled water (control), these parameters recorded a slight increase of 10% and 7% respectively, compared to treated wastewater.

## Discussion

The present study reported microbial synthesis, characterization, and application of manganese nanoparticles (Mn-NPs) prepared by using a bacterial strain, namely *B. flexus*. The brown color of the synthesized nanoparticles was caused by surface plasmon resonance, which was induced by the oscillation of electrons on the nanocomposite’s surface (Wadhawan et al., 2020). The absorption spectra of MnO-NPs illustrate a sharp peak at 325.23nm due to the transmission of electrons from the valance band to the conduction band (**Figure 2**). The absorption spectral test was performed, and it complied with the result performed by Jayandran et al. (2015), whose absorption peak was in the range of 350-410 nm. The findings from the XRD analysis exhibited diffraction peaks at 101, 201, and 210, indicating the crystalline structure of MnO-NPs (**Figure 3**). Similar results for the MnO-NPs were reported by Moon et al. (2015). **Figure 5** illustrates the FTIR spectrum of MnO-NPs at a broad peak of 3397.55 cm^-1^, 2958.31 cm^-1^, 2925.46 cm^-1^, and 1725.41 cm^-1^. The peak at 3394 cm^-1^ was confirmed by the OH group, which is parallel to Saod et al. (2022), in which the OH and CH groups were confirmed at peaks of 3360 cm^-1^ and 2800 cm^-1^.

Biosynthesized manganese nanoparticles were found to be efficient in wastewater treatment. In the present study, MnO-NPs showed great potential for the treatment of wastewater. The biosynthesized MnO-NPs at the concentration of 1g L^-1^ were used for the photocatalytic degradation and adsorption of phosphates, sulphates, COD, TDS, and color intensity of wastewater. Maximum results were gained at the concentration of 1g L^-1^ for the treatment of wastewater under sunlight for seven hours which was also previously reported by (Islam et al., 2018). The result was also positively correlated with Jain et al. (2022) in which they used MnO-NPs at 1mg L^-1^ for the treatment of wastewater. MnO-NPs play a crucial role in the decolorization and degradation of pollutants in wastewater (Cui et al., 2015; Shoiful et al., 2020). When exposed to light, they generate electron-hole pairs, which subsequently interact with oxygen and water (Fouda et al., 2021). This interaction leads to the formation of highly reactive species, such as hydroxyl radicals (Wang et al., 2020). These radicals are responsible for the effective decolorization and degradation of pollutants in the wastewater. These free radicals interact with the contaminants and transform them into less toxic and simpler molecules (Zhao et al., 2015; Yamaguchi et al., 2018). Gupta et al. (2015) reported that use of nano particles is a useful approach to remove the noxious metal ions from the wastewater as they have specific surface characteristics and unique structure. Nanosized metal oxides, including manganese oxides, possess high surface area and specific affinity along with other mechanisms like electrostatic interaction, ion exchange, surface complexion and hard/soft acid-base interaction to absorb impurities from wastewater.

Results showed the removal of 79% COD, 37% TDS, 64% sulfates, 64.5% phosphate, 77.05% color intensity, pH reduction from 8.7 to 7.3, and EC reduction from 5.33 to 3 (**Table 1**). COD is the parameter against which MnO-NPs show the highest efficiency. MnO-NPs are effective adsorbents for removing pollutants from wastewater due to their higher surface area and higher reactivity, which are caused by their absorption properties (Islam et al., 2018). Various contaminants, such as heavy metals, organic compounds, and dyes, can be adsorbed by MnO-NPs (Martinez-Vargas et al., 2018). Such results have also been reported in previous studies (Shabanloo et al., 2020; Jothinathan et al., 2022; Munir et al., 2023). Results of the experimentare supported by the findings of Yaashikaa et al. (2022), who stated that MnO-NPs can efficiently adsorb heavy metals and other contaminants and ultimately degrading COD TDS, phosphates, sulfates, pH, and EC. The findings of the present studies, like removal of 37% TDS, are in line with the outcomes of Kumar and Chopra (2018), who stated that a reduction in TDS was observed by using phytoremediation for wastewater treatment. The TDS might be removed from wastewater by the process of adsorption due to unique properties NPs, like the high surface area to volume ratio and the large number of active sites that interact with TDS (Hendren et al., 2013).

The results revealed that the growth parameters of wheat were significantly increased in treated wastewater as compared to untreated wastewater. The germination rates observed in treated wastewater were as follows: 87% on day nine, 75% on day six, and 62% on day three (**Figure 6**). The germination rate in treated wastewater jumped due to a variety of factors that affected the sustainability of seeds and growth (Dimkpa et al., 2018). The absence of pollutants or hazardous compounds in treated wastewater can generate a more favorable environment for germination as compared to untreated wastewater (Kannan and Upreti, 2008; Huma et al., 2012). The greater germination rates may be due to improved quality of water and nutrient availability in treated wastewater (Nsiah-Gyambibi et al., 2022). Results obtained through the current experiment are supported by Helaly et al. (2018), who concluded that an increase was found in shoot and root lengths of mango plants by the application of treated wastewater. The application of wastewater adversely affected the plant’s primary root elongation, lateral root formation, and growth of aerial parts. Biochemical compounds, like chlorophyll pigments, were decreased in the mesophyll cells by the application of wastewater (Hong et al., 2019).

Wastewater disturbs the physical and physicochemical system of wheat due to the high concentration of COD, TDS, and nutrients (Zhang et al., 2021). The high amounts of phosphate and nitrate disturb metabolism by unbalancing the nutritional system of plants and accumulating in cells and tissues (Ahmad et al., 2020). The chlorophyll parameters of wheat decreased considerably under wastewater treatment as compared to treated wastewater (**Figure 8**). The pollutants present in wastewater reduced oxygen availability by contaminating the soil around wheat, thus reducing physical growth (Khan et al., 2009). The result revealed that the chlorophyll content of wheat increased with the application of treated wastewater, and the health of any plant is depicted by its chlorophyll content (Kamble et al., 2015). Prior research found that treating wheat plants with Mn salt, increased the formation of chlorophyll (Sheng et al., 2016), however, excessive Mn exposure (378 mg/L) decreased chlorophyll concentration (Jhanji et al., 2014). It is well known that Mn is needed in the shoots (leaves) for the functions of chlorophyll and photosynthesis, depending on the effects of soil versus foliar exposure (Narwal et al., 2012). By adding nanoparticles to treat the wastewater, which contains vital nutrients like nitrogen and phosphorus needed for the synthesis of chlorophyll (Khan et al., 2009). The outcomes of the current study are in line with the findings of Jhanji et al. (2014), they reported a consistent outcome, whereby the application of treated wastewater resulted in a significant increase in chlorophyll content in wheat plants.

The results showed that wheat seedlings irrigated with wastewater have higher concentrations of zinc and copper in their roots and shoots than wheat seedlings irrigated with treated wastewater. In industrial discharges, wastewater may include higher quantities of Zn and Cu (Tariq et al., 2020). When such wastewater was used for irrigation, these pollutants may accumulate in the soil and, as a result, in the plant parts. Manganese oxide nanoparticles in treated wastewater may have absorbed or immobilized some of Zn and Cu ions present in the wastewater (Islam et al., 2018). This can make these elements less available for plant absorption, resulting in lower concentration of these elements in plant parts (Zwolak et al., 2019). Wheat seedlings irrigated with treated wastewater have higher levels of manganese and iron than irrigation with distilled water. Manganese oxide nanoparticles have the potential to boost manganese solubility and bioavailability in water, making them more available for plant absorption (Gillispie et al., 2019).

The findings of the study indicated that the application of treated wastewater resulted in an augmentation of antioxidants activities in wheat (**Figure 9**). The previous observation of Hashmat et al. (2021) that higher levels of antioxidant activities predominated in plants kept in under-treated wastewater applications was confirmed by a considerable rise in SOD and POD activities in treated plants. The antioxidants operate effectively as a cooperative network because of a series of redox reactions (Ali and Alqurainy, 2006). It is plausible to assume that enhanced enzymatic activities were caused by higher production of H_2_O_2_ in a water-scarce environment (Ahmad et al., 2019; Faria et al., 2020). An ecological response for plants in stressful settings may be provided by the increase in CAT and POD activities in water-stressed plants (Panyuta et al., 2016). Plant species with poor POD activity may find it difficult to cope with stress because lipid peroxidation reduces the permeability of their membranes (Zarinkoob et al., 2021). During stress, reactive oxygen species (ROS) like H_2_O_2,_ and MDA are produced (Das and Roychoudhury, 2014). Results revealed that ROS decreased in treated wastewater as compared to untreated wastewater. H_2_O_2_ reacts with lipid membranes and damages the permeability of the cell (Javed et al., 2017).

The higher MDA and H_2_O_2_ concentrations were probably caused by oxidative stress induced by the higher absorption of hazardous wastewater components, affecting plant development and yield (Dudziak et al., 2019). It was hypothesized that elevated levels of lipid peroxidation and H_2_O_2_ would result in reduced cellular membrane functions due to heavy metal interference and absorption with mineral nutrients in fertigated soil wastewater (Hashmat et al., 2021).

Furthermore, treated wastewater has beneficial potential for the reduction of various plant parameters, such as MDA and H_2_O_2_. Moreover, it significantly (*p ≤* 0.05) enhanced the growth and antioxidant parameters. According to the findings of Kanwal et al. (2020), physiological and biochemical attributes, including photosynthetic rate, respiration rate, stomatal conductance, chlorophyll, and carotenoids were significantly reduced by the application of wastewater. Application of wastewater as irrigational water resulted in a significant reduction in the morphological, physiological, anatomy, and yield parameters of wheat cultivars (Hajihashemi et al., 2020).

Based on the finding of the study, it is suggested that treatment of wastewater through manganese oxide nanoparticles hold significant implications for agricultural practices, particularly in regions grappling with water scarcity and wastewater management challenges. By demonstrating the effectiveness of MnO-NPs in enhancing both wastewater treatment and wheat seedling growth, the research suggests a potential solution for sustainable agriculture in water-stressed areas. Implementing this technology could lead to more efficient utilization of limited water resources, as treated wastewater could be safely reused for irrigation. Additionally, the antioxidant properties of MnO-NPs could bolster plant resilience to environmental stressors, potentially leading to higher crop yields. Based on the utilization of MnO-NPs in treating wastewater for agricultural purposes, several practical recommendations can be offered for farmers and policymakers. It is advisable to explore their incorporation into wastewater treatment strategies for agricultural irrigation by considering the effectiveness of MnO-NPs in enhancing wastewater quality and promoting plant growth. It can lead to more sustainable water use practices, particularly in regions facing water scarcity. Additionally, the study underscores the importance of carefully monitoring the concentrations and application methods of MnO-NPs to ensure their optimal and safe utilization in agricultural settings. However, it is crucial to acknowledge potential limitations and risks, such as the need for thorough assessments of long-term environmental impacts, including effects on soil health and microbial communities.

## Conclusion

It can be concluded that the utilization of manganese nanoparticles (MnO-NPs) has a potential to treat wastewater by lowering sulfates, phosphates, color intensity, pH, electrical conductivity, total dissolved solids, and chemical oxygen demand. Furthermore, treated wastewater positively influenced the growth parameters, physiological parameters, and antioxidant defense systems of wheat, except malondialdehyde, and H_2_O_2_. Based on the findings, it is concluded that treated wastewater can be effectively utilized to cultivate wheat crop under limited availability of good quality water.

## Data availability statement

The raw data supporting the conclusions of this article will be made available by the authors, without undue reservation.

## Author contributions

AI: Conceptualization, Data curation, Methodology, Writing – original draft. MS: Conceptualization, Data curation, Project administration, Supervision, Writing – original draft. MN: Methodology, Resources, Writing – review & editing, Investigation, Project administration. DI: Methodology, Writing – original draft, Data curation, Formal analysis, Resources, Writing – review & editing. SH: Conceptualization, Data curation, Formal analysis, Writing – original draft. TS: Formal analysis, Writing – original draft, Investigation, Methodology. FM: Investigation, Conceptualization, Software, Writing – review & editing. AR: Conceptualization, Investigation, Formal analysis, Resources, Writing – original draft. SG: Conceptualization, Data curation, Methodology, Supervision, Writing – review & editing. A-RG: Conceptualization, Methodology, Writing – review & editing, Investigation, Project administration, Resources. MH: Formal analysis, Methodology, Resources, Writing – review & editing, Software. SK: Conceptualization, Methodology, Resources, Writing – review & editing, Validation, Writing – original draft.
